# Polarization of Macrophages in Granulomatous Cutaneous T Cell Lymphoma Granulomatous Mycosis Fungoides Microenvironment

**DOI:** 10.3390/dermatopathology9010009

**Published:** 2022-02-25

**Authors:** Lopez Dominguez Johanny, Olayemi Sokumbi, Misty M. Hobbs, Liuyan Jiang

**Affiliations:** 1 Mayo Clinic Alix School of Medicine, Jacksonville, FL 32224, USA; lopezdominguez.johanny@mayo.edu; 2 Department of Dermatology, Mayo Clinic, Jacksonville, FL 32224, USA; sokumbi.olayemi@mayo.edu (O.S.); hobbs.misty@mayo.edu (M.M.H.); 3 Department of Pathology and Laboratory Medicine, Mayo Clinic, Jacksonville, FL 32224, USA

**Keywords:** mycosis fungoides, granulomatous mycosis fungoides, macrophage polarization, tumor microenvironment, tumor associated macrophages

## Abstract

Polarization of tumor associated macrophages (TAMs) has been shown to have prognostic significance in different cancer types. This study evaluates the macrophage subtypes that predominates in GMF. Cases of GCTCL from 2007–2020 were identified (*n* = 6), clinical data was extracted from the electronic medical record, and all pathology slides were reviewed to confirm the diagnosis. Immunohistochemistry (IHC) studies were performed to characterize M1 and M2 macrophage polarization. CD68 (PGM1), pSTAT1, and CD163 were used as pan macrophage, M1, and M2 markers, respectively. The macrophages with positive staining at hot spot per high power field were counted and recorded for data analysis. The average age of patients was 60.5 years [range, 21–78], five patients (83%) were women and 1 (17%) was a man. Five patients were Caucasian (83%), and 1 was Black/African American (17%). Two patients had late stage GMF with M2 (CD163) predominance and the other three had early stage GMF with M1 (pSTAT1) predominance. Our study suggests that macrophage polarization present in GMF tends to be M1 in early stages and M2 in advanced stages. Additional studies are needed to further elucidate the microenvironment of macrophages present in GMF. Such findings may lead to prognostic and therapeutic advances in GMF.

## 1. Introduction

Granulomatous cutaneous T-cell lymphoma (GCTCL) represents a rare variant of cutaneous lymphomas, accounting for only 2% of all cases [1,2]. First described in 1970 by Ackerman and Flaxman, it is characterized histologically by a predominantly histiocytic and/or granulomatous infiltrate that often obscures the underlying malignant lymphocytes, therefore often leading to misdiagnosis as well as delay in diagnosis and treatment [3,4,5]. GCTCL presents a diagnostic challenge due to its rarity and clinicopathological heterogeneity. While the granulomatous slack skin variant of GCTCL is a distinct clinical subtype with bulky pendulous skin folds, the granulomatous mycosis fungoides (GMF) subtype has no distinct clinical presentation [4,5].

The exact pathogenic mechanisms of granuloma formation in GCTCL remains poorly understood. Specifically, there is limited data describing the microenvironment composition of granulomas in GCTCL. Macrophages present in granulomas are polarized into either a pro-inflammatory (M1) or an anti-inflammatory (M2) subtype. M1 macrophages are associated with a cytotoxic TH1 response and secrete reactive oxygen species that have microbicidal, inflammatory, and anti-tumor functions [6]. Conversely, M2 macrophages are associated with an immunoregulatory TH2 response responsible for the secretion of growth factors and inhibition of cell death pathways, which can shield tumor cells from the effects of chemotherapy [6]. Prior studies have associated a higher M1/M2 ratio with a favorable outcome and better response to treatment when compared to a lower M1/M2 ratio in pediatric classical Hodgkin Lymphoma, ovarian cancer, locally advanced cervical cancer, breast cancer, lung cancer, hepatocellular carcinoma, and gastric cancer [6]. While the M1/M2 ratio of tumor associated macrophages (TAMs) has been shown to have prognostic significance in different cancer types with a prominent granulomatous component, similar studies are lacking in GCTCL [6,7]. 

Herein, we aim to characterize the TAMs that predominate in GCTCL to better understand the significance of this histologic finding. 

## 2. Materials and Methods

This study was approved by the Mayo Clinic Institutional Review Board. Within a thirteen-year period from 2007 to 2020, a total of six cases with a definitive diagnosis of granulomatous cutaneous T-cell lymphoma (GCTCL) were identified. 

Clinical information was extracted from the electronic medical record including age, gender, race, date of presentation at our practice, date of GCTCL diagnosis, lesion morphology, clinical images, treatment and follow up data. All available pathology slides and ancillary studies including molecular study by polymerase chain reaction for T cell receptor gene rearrangement were reviewed by a dermatopathologist (OS) and hematopathologist (LJ) with expertise in the diagnosis of cutaneous lymphomas. Histological features were assessed including predominant patterns, composition of cellular infiltrates, distribution and density of the atypical lymphocytic infiltrates, and dermal stromal changes. 

Besides the diagnostic immunostaining markers (CD3, CD20, CD4, CD5, CD7, CD8), additional immunohistochemistry (IHC) studies were performed on Leica and Ventana platforms to characterize M1 and M2 macrophage polarization. CD68 (PGM1) (clone PG-M1, DAKO), pSTAT1 (clone 58D6, Cell Signaling), and CD163 (clone EPR19518, Abcam) were used as pan macrophage, M1, and M2 markers, respectively. Appropriate positive and negative controls were used to ensure the quality of IHC stain and antibody titration. PGM1, pSTAT1, and CD163 immunostaining slides were reviewed by the hematopathologist (LJ); the macrophages with positive staining at hot spot per high power field were counted and recorded for data analysis. 

Despite the small sample size for any significant biostatistical conclusion, we summarized the variables to describe the pattern of association between M1–M2 difference and patient age, race, and stage. Categorical variables were summarized as frequency (percentage) and continuous variables were reported as median (range). M1 predominance is defined as M1 positive cells more than M2 with a difference of ≥10 cells/HPF; and M2 predominance is defined as M2 more than M1 with a difference of ≥10 cells/HPF. Stage I to IIa was grouped as early stage mycosis fungoides, and stage IIb to IV as advanced stage mycosis fungoides. The analysis was done using R3.6.2. 

## 3. Results

### 3.1. Clinical Features

A summary of the clinical features in the six GCTCL patients studied are reviewed in Table 1. Five patients (83%) were women and one (17%) was a man. The average age was 60.5 years [range, 21–78]. Five patients were Caucasian and one was Black/African American. Three patients presented with early stage GCTCL (Stage Ia-IIa) and three with advanced disease (stage IIb-IVa). The lesion morphology varied and included macules (2/6 cases), patches (5/6 cases), plaques (2/6 cases), and papules (1/6 cases). Distribution of lesion included generalized with involvement of the face (3/6), trunk, arms, and legs (2/6), and trunk and legs (1/6). Patients received a variety of standard therapies including topical corticosteroids, topical retinoids, methotrexate, imiquimod, radiation therapy, and phototherapy. The mean follow-up time after presentation was 7 years. One patient was lost to follow up (patient 1). This patient was recommended CHOP therapy by an outside hematologist/oncologist; however, treatment completion status and outcome data are not available. One patient (patient 5) died with their disease; no data is available regarding the cause of death. This patient had a progressive disease course despite trying six different therapies over the course of four years. Only one out of the six patients achieved complete tumor regression, and three out of six experienced disease progression despite being on multiple treatment modalities. 

### 3.2. Pathological Features

**Figure 1:**
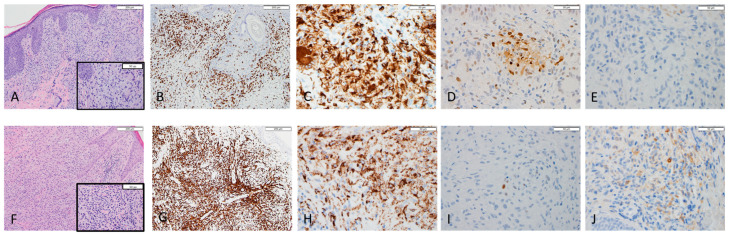
Case #2 skin biopsy with M1 predominancy (**A**–**E**); the biopsy demonstrates dense dermal proliferation of mixed cells including neoplastic lymphocytes and abundant histiocytes; many multi-nucleated giant cells are seen (**A**, inset × 40). Immunostain of CD3 shows interstitial infiltraTable 68. highlights histiocytes and multi-nucleated giant cells (**C**); pSTAT1 is positive for a cluster of M1 macrophage (**D**) while CD163 for M2 is completely negative I. Case #3 skin biopsy with M2 predominancy (**F**–**J**); the biopsy demonstrates tumor stage of mycosis fungoides. The neoplastic T lymphocytes is in a background rich in histiocytes and eosinophils (**F**, inset × 40). Immunostain of CD3 shows diffusely infiltration of neoplastic T lymphocytes (**G**); CD68 is positive for the histiocytes (**H**). pSTAT1 highlights rare positive M1 macrophages (**I**), while CD163 shows increased M2 macrophages (**J**).

Histopathologic and immunohistochemical findings in cutaneous specimens studied are presented in Table 1. Representative images of cases #2 and #3 are shown in Figure 1. Histologic patterns observed consisted of diffuse (2/6), perivascular (2/6), interstitial (1/6), and folliculotropic (2/6) infiltrate. Immunohistochemistry of all pathology slides was positive for PGM1 marker with cytoplasmic staining pattern confirming the presence of a histiocytic and/or granulomatous response in all cases evaluated. There are two patients (case #1 and #3) with M2 predominance (positive for CD163 with cytoplasmic stain) and the other three (case #2, #4, and #6) with M1 predominance (positive for pSTAT1 with nuclear stain). One case (case #5) has both M1 and M2 cells less than 10/HPF; therefore, we have eliminated that case from the final statistical analysis. The clinical correlation with macrophage polarization is summarized in Table 2. 

## 4. Discussion

Macrophages are present in most tissues as professional phagocytic and immunomodulating cells essential for maintaining host homeostasis. Their role includes antimicrobial activity against various pathogens, tissue repair, and even tumor modulation [8]. Macrophages exist in tissues as a heterogenous cell population influenced by their environment and differing in their response to inflammatory cytokines. In the early 2000s, Mills et al. proposed the polarization of macrophages into M1 and M2 [9]. M1 polarized macrophages, also known as classically activated macrophages, are described as pro-inflammatory with a role in pathogen resistance and tissue destruction [9]. M1 macrophages are activated by INF y, lipopolysaccharide (LPS, a component of the outer membrane of bacteria), and granulocyte macrophage colony stimulating factor (GCSF [6]. On the other hand, M2 polarized macrophages, also known as alternatively activated macrophages, are anti-inflammatory and respond to IL-4 and TGF-B1 [6]. M2 macrophages have a role in host immune response against parasites and in repair after acute tissue damage [6]. 

Tumor associated macrophages (TAMs) has also been described as having an important role in tumor modulation [9]. TAMs that exhibit an M2 phenotype are linked to tumor proliferation, invasion, metastasis, and inhibition of anti-tumor immune response of T cells. [9]. A recent meta-analysis of Non-Hodgkin lymphoma found that the type of TAMs found within a tumor microenvironment served as a prognostic indicator [10]. Tumors with high density of CD163+, a specific marker for M2 polarized macrophages, were associated with lower overall survival (OS) and a low progression-free survival (PFS) [10]. Increased number of CD163+ cells and serum soluble CD163 were observed in CTCL, atopic dermatitis, and psoriasis comparing with normal skin; increased lesion macrophages also been associated with disease progression and an overall worse prognosis in CTCL [11]. Moreover, although the exact mechanisms remain unknown, chemotherapy agents targeted on TAMs via reprograming M2 macrophages or promoting antitumor activities have proven effective in various hematopoietic malignancies including multiple myeloma and CNS lymphomas [12,13,14,15]. 

While several tumors with a high density of M2 polarized macrophages have been associated with worse prognosis, there are no studies in the literature that examine the macrophage polarization in granulomatous mycosis fungoides. The GMF cases studied here showed a predominant M2 macrophage polarization in 2/6 cases and M1 macrophage predominant in 3/6 cases. All cases with M1 polarization had an early disease stage, whereas cases with M2 polarization had advanced disease. Our study found that M2 polarization was only present in patients with more advanced disease stage. One possible explanation is that, as seen in previously reported malignancies, patients with an M2 predominance have a more aggressive disease course [6,10]. As such, M1 macrophages in GMF likely represent a pro-inflammatory response that leads to suppression of T-cell lymphoma. Another possibility is that in the early stages GMF might be associated with a pro-inflammatory M1 infiltrate that is then altered into an anti-inflammatory, pro-tumor M2 response as the disease progresses. More studies are needed to further explore this relationship and elucidate the prognostic value of macrophage polarization. Larger studies are needed to examine how disease progression might differ in patients with M1 versus M2 predominance. 

We found no relationship between patient’s age and macrophage predominance. Additionally, patients presented with varied lesion location and morphology, which was not related to the type of macrophage predominance observed within the granulomas. No relationship was observed between gender or race and macrophage predominance. 

This study is limited by the small number of cases given the rarity of GMF. Larger studies are needed to understand how the microenvironment found within the infiltrate of GMF might have important implications for the treatment and prognostication of this condition. In addition, future development of treatment for GMF might focus on targeting the microenvironment of the tumor to either accentuate or attenuate the M1 or M2 response, respectively. 

## 5. Conclusions

Our study suggests that macrophage polarization present in GMF tends to be M1 in early stage and M2 in advanced stages. Additional studies are needed to further elucidate the microenvironment of macrophages present in GMF and to learn how they impact the progression of disease. Such findings will lead to important prognostic and therapeutic advances in GMF. 

## Figures and Tables

**Table 1:** Clinical, histologic, immunophenotypic, and treatment data of 6 patients with granulomatous mycosis fungoides.

Patient No./Sex/Race/Age, y	Lesion Location	Lesion Morphology	TNM Stage	Histologic Pattern	Predominant Macrophage Polarization	Therapy	Treatment Response	Outcome/ Follow up, y
1/F/BAA/21	Generalized	Macules (exfoliative erythrodermatous and skin colored)	IVa	Diffuse	M2	CHOP	LTF	NA, LTF
2/F/C/25	Generalized	Macules, patches and plaques (purpuric, annular scaling)	IIa	Diffuse, Perivascular	M1	CS, MTX	PR	AWD, 1
3/F/C/75	Trunk, arms, legs	Patches (scaly erythematous)	IIB	Perivascular	M2	CS, ret, UVB, imiq	PR	AWD, 22
4/F/C/51	Trunk, arms, legs	Patches (erythematous)	IB	Interstitial	M1	Elidel, UVA, MTX	PR	AWD, 6
5/M/C/70	Generalized	Patches and plaques	IIb	Folliculotropic	Not applicable due to low cell count	CS, MTX, UVB, RT, ret, imiq	PD	DWD, 4
6/F/C/78	Trunk, legs	Papules and patches (scaling)	Ia	Folliculotropic	M1	CS, PUVA, ret	CR	ACR, 2

Abbreviations: ACR, alive with complete remission; AWD, alive with disease; BAA, black/African American; C, Caucasian; CR, complete tumor regression; CS, topical corticosteroids; DWD, died with disease; F, female; imiq, imiquimod; LTF, lost to follow up; M, male; MTX, methotrexate; NA, not available; PD, progressive disease; PR, partial tumor regression; PUVA, psoralen ultraviolet A phototherapy; ret, retinoids; RT, Radiation therapy; UVA, ultraviolet A phototherapy; UVB, ultraviolet B phototherapy.

**Table 2:** Demographic and stage data by M1–M2 difference.

	M2 Predominant	M1 Predominant	Total (*n* = 5)
Age			
*n*	2	3	5
Median (Range)	48.8 (21.0, 75.0)	51.0 (25.0, 78.0)	50 (21.0, 78.0)
Race			
African American	1 (50.0%)	0 (0.0%)	1 (20.0%)
Caucasian	1 (50.0%)	3 (100.0%)	4 (75.0%)
Stage			
Early	0 (0.0%)	3 (100.0%)	3 (60.0%)
Advanced	2 (100.0%)	0 (0.0%)	2 (40.0%)

## Data Availability

Data is available upon reasonable request.
